# Microscopic Primary Tubal Serous Carcinoma With Colon Metastasis and Paraneoplastic Neurological Syndrome: A Case Report

**DOI:** 10.1002/ccr3.71131

**Published:** 2025-10-04

**Authors:** Tyler Ye, Dinuke De Silva, Leonardo D. Santos, Felicia Roncolato, Stanley Levy, Auerilius Erastus Ricardo Hamilton

**Affiliations:** ^1^ School of Medicine Western Sydney University Campbelltown New South Wales Australia; ^2^ Department of Anatomical Pathology Liverpool Hospital Liverpool New South Wales Australia; ^3^ Department of Medical Oncology Campbelltown Hospital Campbelltown New South Wales Australia; ^4^ Department of Neurology Campbelltown Hospital Campbelltown New South Wales Australia; ^5^ Department of General Surgery Campbelltown Hospital Campbelltown New South Wales Australia

**Keywords:** anti‐Yo antibody, colon biopsy, colonic metastasis, fallopian tube neoplasm, high‐grade serous carcinoma, paraneoplastic neurological syndrome

## Abstract

This case report discusses a rare instance of a 69‐year‐old woman presenting with lower limb paresthesia, whose imaging showed suspicious nodules in the sigmoid colon and no other obvious sites of malignancy. Laparoscopic anterior resection was performed and subsequent immunohistochemical examination revealed an unexpected diagnosis of colonic metastasis from a carcinoma of tubo‐ovarian origin, which necessitated a second surgery. Metastatic lesions of the colon from other organs are uncommon, and accurate diagnosis without clear evidence of the primary tumor poses a challenge. This case emphasizes the consideration for re‐biopsy when initial samples lack sufficient tissue for thorough immunohistochemical examination, highlighting the importance of detailed histopathology in guiding diagnosis and treatment in metastatic cases. We also discuss the utility of identifying paraneoplastic neurological syndromes and onconeural antibodies in determining the tubo‐ovarian origin of cancer.

**Summary**

- Colonic metastasis from primary fallopian tube carcinoma is rare and can mimic primary colorectal cancer.
- This case highlights the diagnostic challenge and underscores the importance of considering paraneoplastic neurological syndromes as an initial presentation of an occult gynecological malignancy.

## 1 Introduction

Secondary metastatic lesions to the colorectum are infrequent, occurring in approximately 1% of all colorectal neoplasms [1]. Primary fallopian tube carcinoma is an uncommon tumor, accounting for approximately 0.14%–1.8% of female genital malignancies [2]. Reports of colon metastases originating from the fallopian tube are limited in the literature [3, 4, 5, 6]. Notably, these instances typically involved patients with a previous history of known malignancy, as well as clinical or radiological findings consistent with recurrent disease [3, 4, 5]. Paraneoplastic neurological syndromes can manifest as the initial presentation of an underlying malignancy. These syndromes arise from an autoimmune response targeting antigens shared between neuronal tissues and tumor cells [7, 8, 9]. Herein, we present a unique case of high‐grade serous carcinoma (HGSC) of the fallopian tube with colon metastasis, initially presenting as a paraneoplastic neurological syndrome.

## 2 Case History and Examination

**FIGURE 1:**
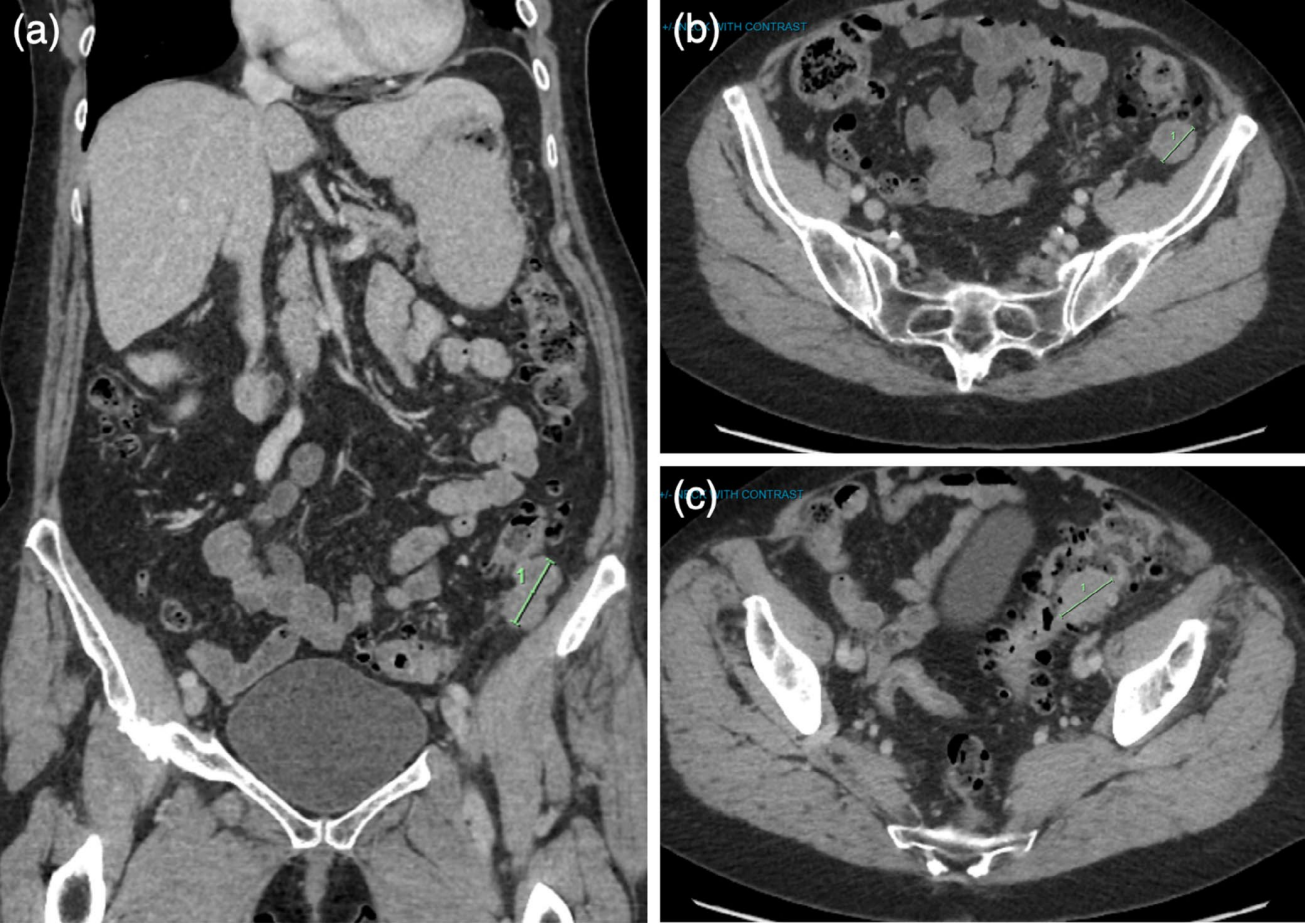
CT images. (a) A bilobed solid lesion was seen within the pericolic fat posteriorly abutting the sigmoid colon. The lesion extended over a length of 38 mm in the coronal plane. (b) The solid lesion measured 25 mm in the axial plane. (c) Focal ovoid area of soft tissue thickening within the sigmoid colon at the left iliac fossa. The lesion involved the colonic wall and measured approximately 35 mm.

A 69‐year‐old woman with an 18‐month history of lower limb paresthesia was referred to a neurology clinic for investigation. She had a past medical history of hypothyroidism, diverticular disease, anxiety, and asthma, and had a previous right ovarian cyst resection at age 30. The patient had a family history of pancreatic cancer in one first‐degree relative and colorectal cancer in another first‐degree relative. Electromyography and nerve conduction studies showed predominantly sensory demyelinating peripheral neuropathy and bilateral common peroneal neuropathy at the level of the fibular neck. Autoimmune serology was positive for anti‐Yo antibodies and anti‐Ro52 antibodies, which prompted investigation for a paraneoplastic etiology by means of computed tomography (CT) of the chest, abdomen, and pelvis (CAP) (Figure 1). CT showed thickening within the sigmoid colon and a separate solid lesion, which was suggestive of a primary colonic neoplasm that had metastasized to an adjacent lymph node. Magnetic resonance imaging found no other causes of neurological dysfunction.

## 3 Investigation, Differential Diagnoses and Treatment

**FIGURE 2:**
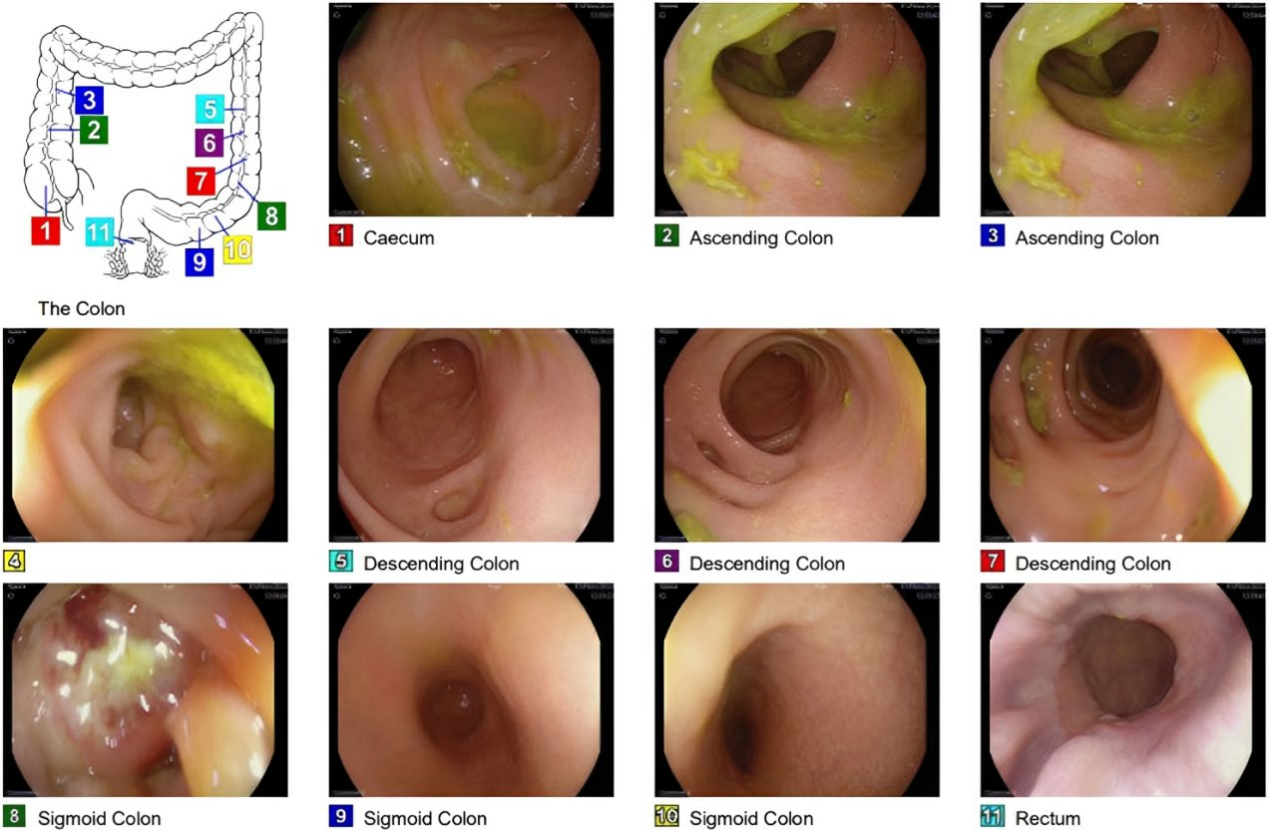
Colonoscopy showing diverticulosis in the descending colon and localized thickening and inflammation in the sigmoid colon.

**FIGURE 3:**
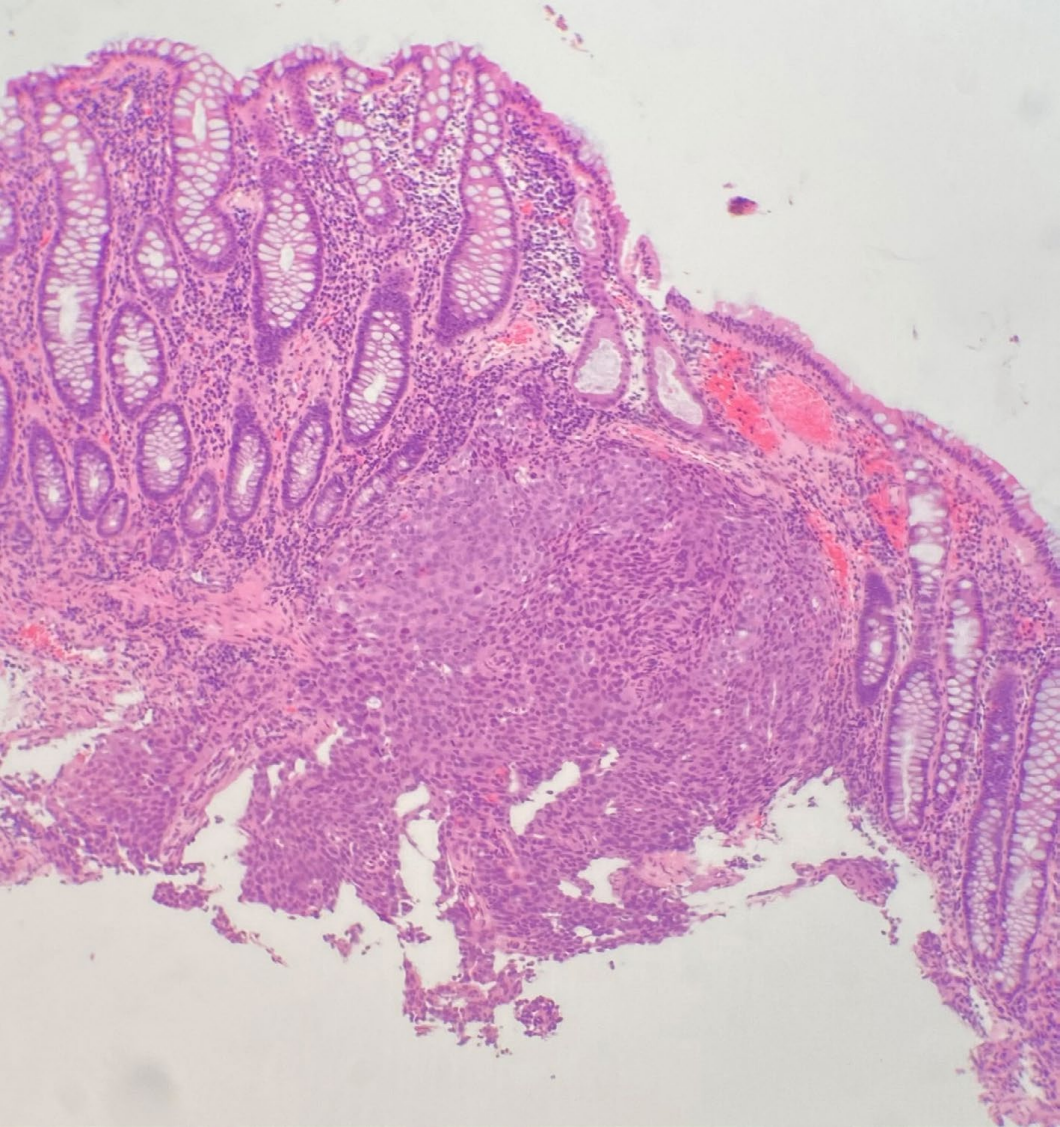
Histological evaluation of the colon biopsy (hematoxylin and eosin, magnification 40×). Within the submucosa, there is a lesional nodule composed of neoplastic epithelioid cells with hyperchromatic nuclei and abundant eosinophilic cytoplasm. The lesion does not involve the overlying mucosa.

**FIGURE 4:**
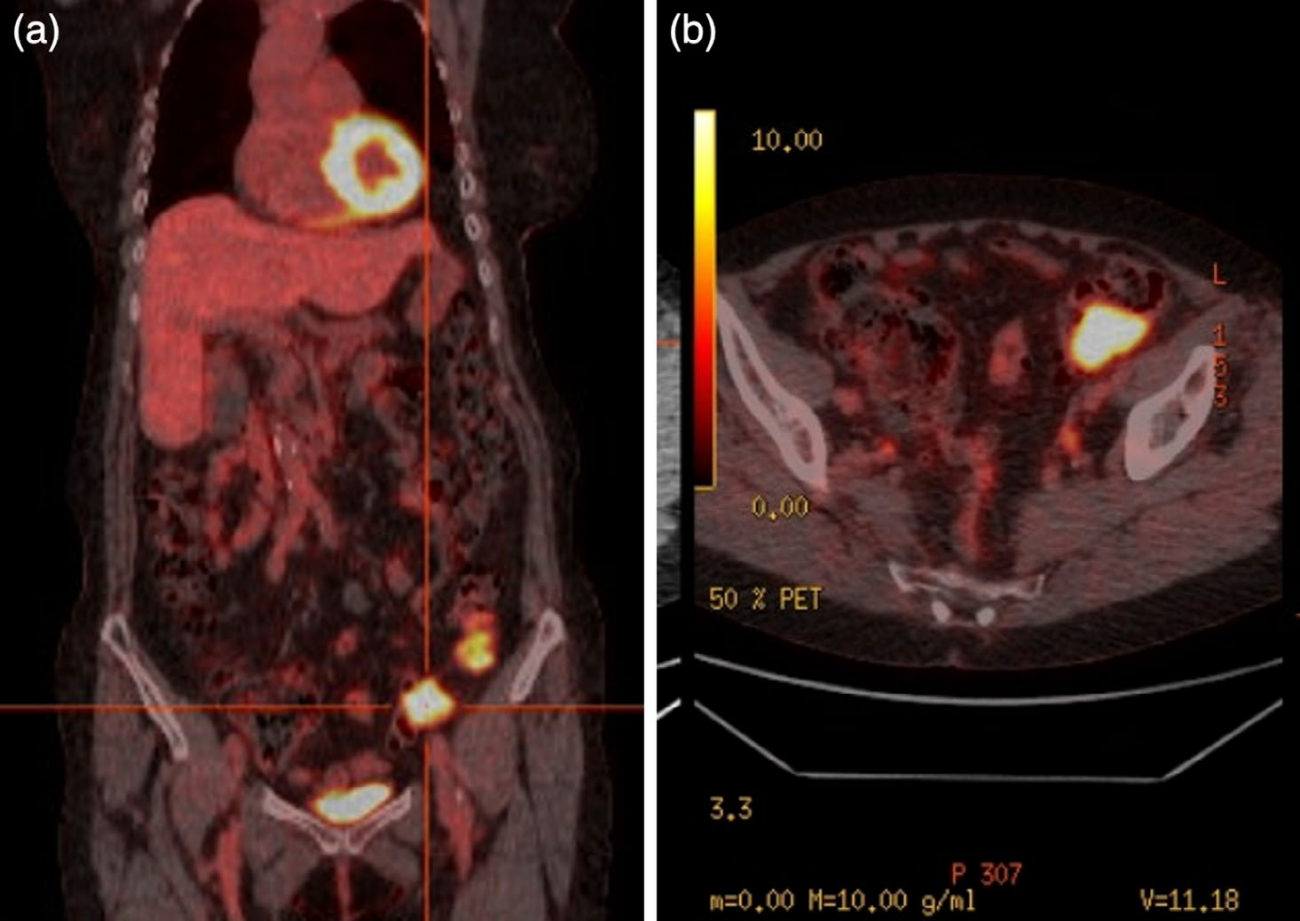
PET Whole Body FDG Study. (a) Coronal view showing two foci of FDG uptake. The crosshairs indicate the location of the soft tissue sigmoid lesion. (b) Axial view showing intense FDG uptake in the soft tissue sigmoid lesion.

The patient was subsequently referred to our hospital based on suspicion of paraneoplastic syndrome associated with primary colon cancer. Blood examination results showed elevated tumor marker levels (carbohydrate antigen 125 = 88 U/mL [reference range: < 35 U/mL] and carbohydrate antigen 19–9 = 69 U/mL [reference range: < 37 U/mL]). A colonoscopy showed localized inflammation and thickening in the sigmoid colon and scattered diverticula in the descending colon (Figure 2). The thickened area in the sigmoid colon was biopsied and microscopic examination revealed a poorly differentiated cancer of unknown origin (Figure 3). Immunohistochemistry (IHC) studies were requested but could not be performed due to insufficient remaining tissue. Positron emission tomography (PET) (Figure 4) demonstrated marked fluorodeoxyglucose (FDG) accumulation in the soft tissue sigmoid lesion and low‐grade activity in the lymph nodes of the cervical, bilateral axillary, and inguinal regions. A separate focus of intense FDG accumulation was seen further proximally, corresponding to an irregular solid lesion abutting the proximal sigmoid and descending colon. There was no abnormal FDG activity to suggest metabolically active malignancy or metastasis elsewhere.

Following multi‐disciplinary discussion, the consensus was that the patient had a paraneoplastic neurological syndrome associated with anti‐Yo antibodies secondary to multiple colonic malignancies. It was unclear at this point whether the lesions were primary or metastatic. Given the diagnostic uncertainty, an extensive workup was required, which accounted for the 6‐month interval between initial presentation and surgery. Immunomodulating therapies such as corticosteroids or intravenous immunoglobulin were not administered, given the limited evidence of benefit in paraneoplastic subacute sensory neuropathy and the prioritization of definitive oncological surgery. Laparoscopic high anterior resection was subsequently performed.

**FIGURE 5:**
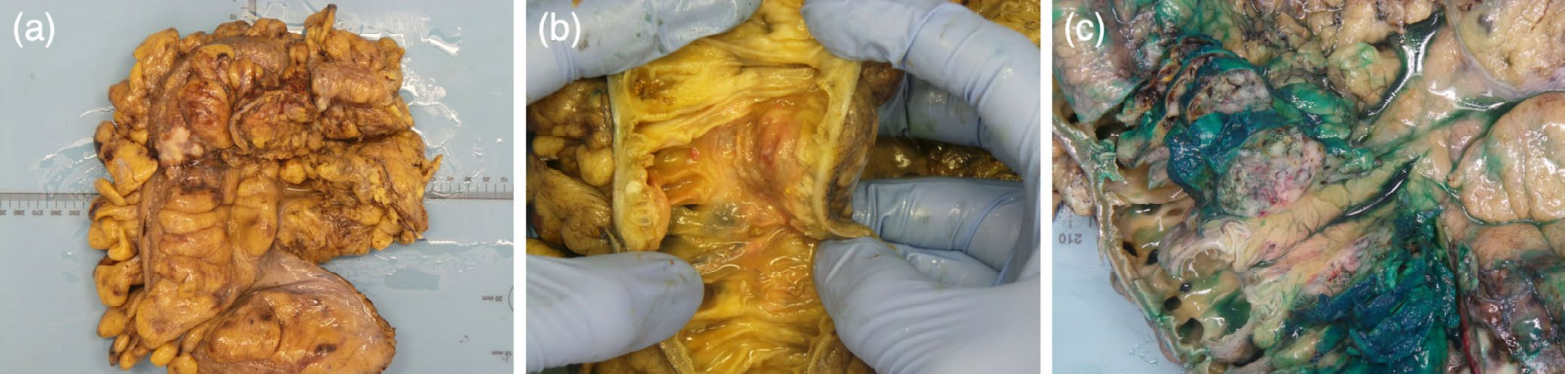
Postoperative evaluation of the colonic resection. (a) Multiple pale lesions seen on serosal surface. (b) Cut surface showing a gray lesion within the bowel wall that is not originating from the mucosa. (c) Nodule located within the pericolic fat.

**FIGURE 6:**
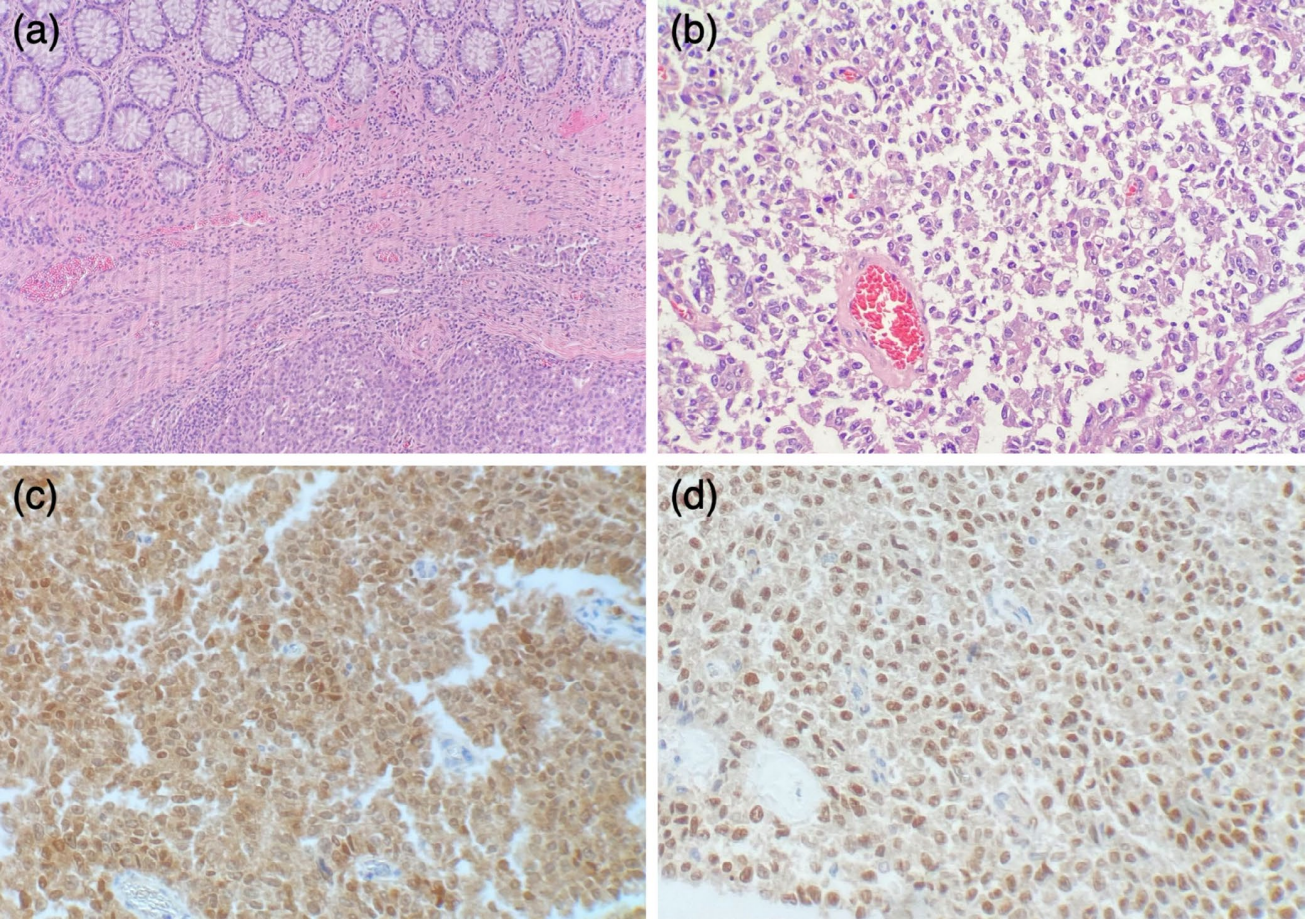
Histological evaluation of the colonic resection. (a) Within the muscularis propria and submucosa, there is an unencapsulated lesion composed of discohesive sheets of epithelioid cells. The lesion does not involve the overlying mucosa (hematoxylin and eosin, magnification 100×). (b) At higher power, the lesional cells contain enlarged, hyperchromatic, pleomorphic nuclei with prominent nucleoli, coarse chromatin, and moderate amounts of eosinophilic cytoplasm (hematoxylin and eosin, magnification 200×). (c) The lesional cells show patchy nuclear positivity for PAX8. (d) The lesional cells show diffuse nuclear positivity for WT1.

At the time of the initial surgery, the ovaries were grossly atrophic, consistent with the patient's age. The resected specimen (Figure 5) revealed a mural tumor measuring 36 mm in maximum dimension and extending into the pericolic fat with multiple, separate, pale, serosal lesions measuring 8 to 45 mm across in maximum dimension. Microscopically, the tumor was centered within the submucosa and muscularis propria and was composed of nodules of atypical, pleomorphic, neoplastic cells surrounded by a desmoplastic stromal reaction with associated necrosis (Figure 6). There was no carcinoma in situ/high‐grade dysplasia of the epithelium. The resection margins were clear (R0) and all 29 pericolic lymph nodes were negative for metastasis. There was no evidence of lymphovascular or perineural invasion. IHC stains showed strong diffuse staining for keratin MNF116 and keratin 7 (KRT7), and strong nuclear staining at the periphery of the carcinoma for PAX8, WT1, estrogen receptor (ER), and progesterone receptor (PR). There was aberrant, strong, diffuse, nuclear staining for p53. The stains for keratin 20 (KRT20), CDX2, SATB2, TTF1, GATA3, chromogranin, synaptophysin, calretinin, keratin 5, keratin 6, and D2‐40 were all negative. These results suggested a HGSC of tubo‐ovarian origin that had metastasized to the colon.

Whilst the location of the potential primary cancer was not confirmed, six cycles of chemotherapy with carboplatin/paclitaxel (dose reduced for her pre‐existing peripheral neuropathy) were administered, with interval debulking, based on histopathology and radiological findings. Interval cytoreductive surgery, which included total laparoscopic hysterectomy, bilateral salpingo‐oophorectomy, and infra‐colic omentectomy, was performed after the 4th cycle of chemotherapy. The right fallopian tube was intraoperatively found to be adherent to the small bowel mesentery.

**FIGURE 7:**
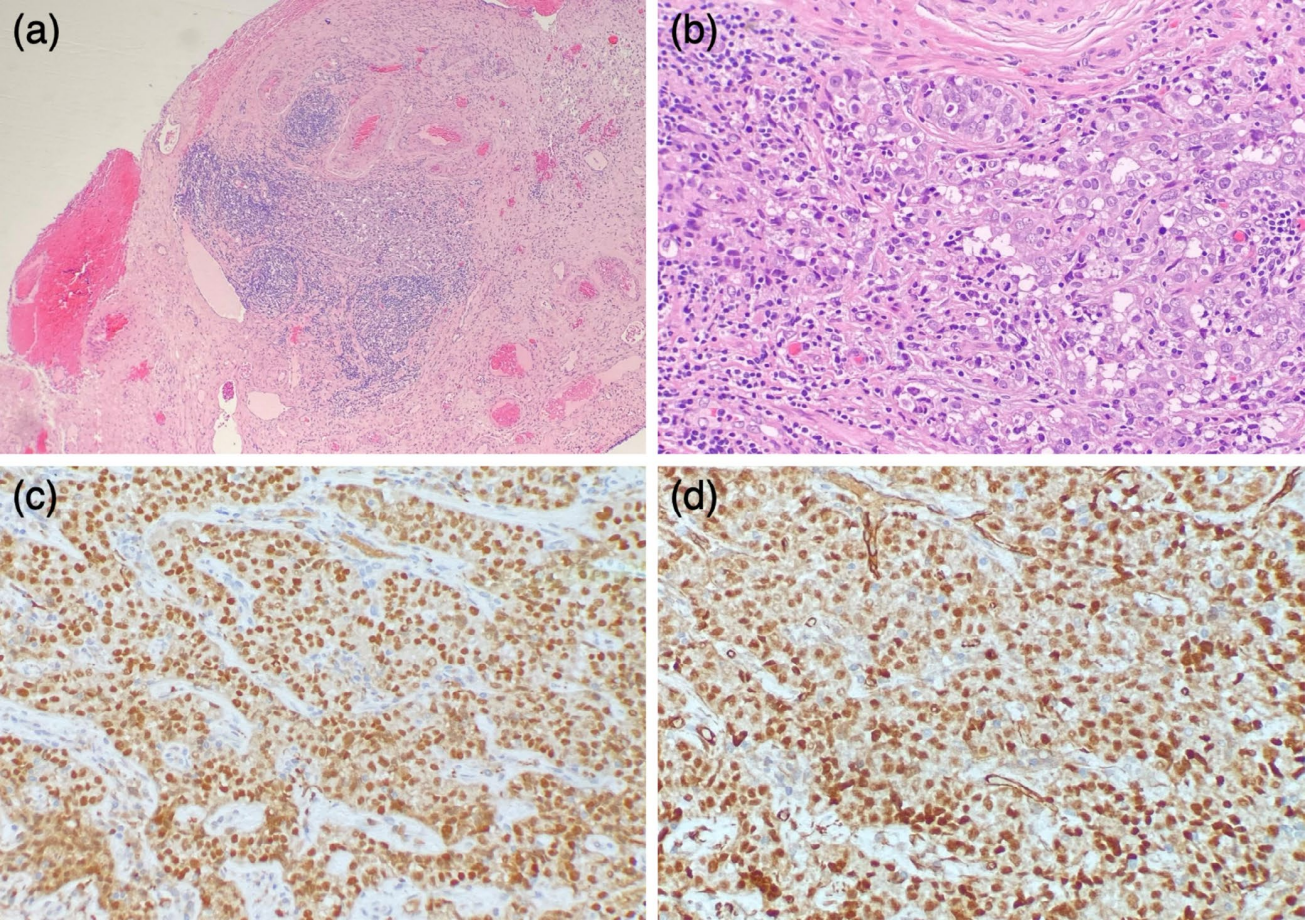
Histological evaluation of the fallopian tube. (a) Sections show the fimbriae of the right fallopian tube containing a well‐circumscribed nodule of lesional cells surrounded by reactive lymphocytes (hematoxylin and eosin, magnification 40×). (b) At higher power, the lesional cells are epithelioid with enlarged, pleomorphic nuclei and abundant clear to eosinophilic cytoplasm (hematoxylin and eosin, magnification 200×). (c) The lesional cells show diffuse nuclear positivity for PAX8. (d) The lesional cells show diffuse nuclear positivity for WT1.

Histopathological examination was conducted on the postoperative specimens using the sectioning and extensively examining the fimbriated end (SEE‐FIM) protocol, which revealed a 1 mm focus of HGSC associated with the right fallopian tube fimbriae (Figure 7). There was no evidence of serous tubal intraepithelial carcinoma or other cancer. IHC staining was positive for keratin MNF116, PAX8, WT1, ER, p53, and p16. There was aberrant, strong, diffuse nuclear staining for p53. Staining for PR was negative. Peritoneal washings were negative for malignant cells.

## 4 Outcome and Follow‐Up

**FIGURE 8:**
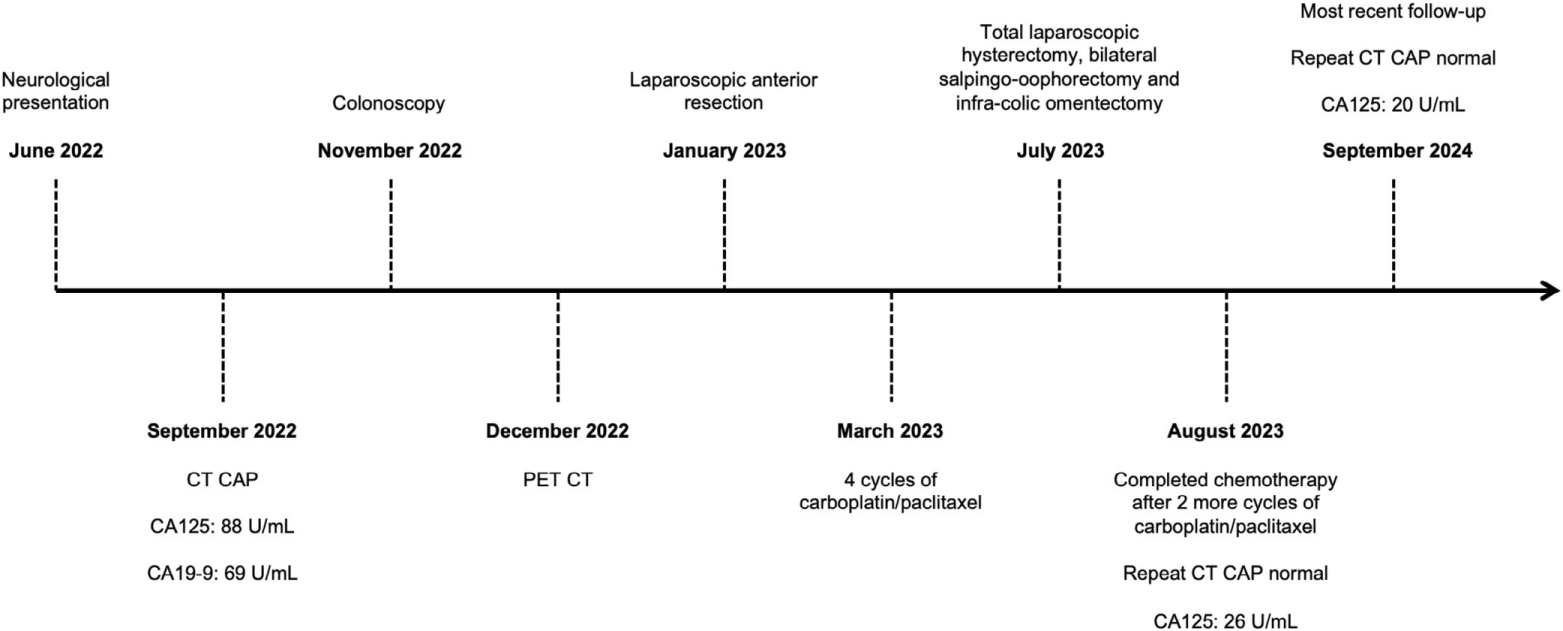
Timeline of the patient's clinical course.

The patient completed chemotherapy 6 weeks after cytoreductive surgery. Subsequent serum tumor markers were normal, and repeat CT CAP and PET scans showed no evidence of disease. Improvement in her neurological symptoms was first noted 4 months after completing chemotherapy. The patient has been followed up every 3 months with an oncologist and periodically with a neurologist. Over the 2‐year follow‐up period since the operation, there has been no evidence of recurrent disease, and the patient's neurological symptoms have significantly improved but remain present. A timeline of the patient's clinical course is shown in Figure 8.

## 5 Discussion

Diagnosis of colonic metastasis from a carcinoma of tubo‐ovarian origin can be challenging, especially when there are no signs of the primary tumor. To our knowledge, this is the only reported case of a primary fallopian tube carcinoma with a colon metastasis presenting with neurological symptoms in the absence of gynecological or gastrointestinal symptoms. The radiological findings of our patient were compatible with primary colon cancer, and the bilateral ovaries and fallopian tubes showed no radiological signs of malignancy, possibly due to the small size of the primary tumor. Usui et al. [6] report the only other case of colon metastasis from tubal HGSC in which the bilateral ovaries and fallopian tubes were radiologically normal.

The initial presentation of our patient was characterized by lower limb paresthesia, which was attributed to a paraneoplastic neurological syndrome. The presence of onconeural antibodies is highly suggestive of an underlying tumor, and if a malignancy is not initially identified, the presence of an occult neoplasm must be assumed [7]. Notably, anti‐Yo antibodies have been described as “high‐risk” and their presence is associated with malignancy in > 90% of cases [7, 8] with the most common cancers being breast and ovarian cancer [8, 9]. Ovarian cancers, in particular, are strongly associated with paraneoplastic neurological syndromes irrespective of antibody type [8, 10], especially subacute cerebellar degeneration and subacute sensory neuropathy [9]. Studies have suggested that most HGSCs originate in the fallopian tubes [11, 12, 13], and thus associations observed in ovarian HGSC can be understood as synonymous to those found in fallopian tube HGSC. Accordingly, paraneoplastic cerebellar degeneration and the presence of anti‐Yo antibodies have been reported in multiple cases of primary fallopian tube serous carcinoma [14, 15, 16, 17, 18, 19]. However, to our knowledge, this is the only reported case of a sensory neuropathy in the context of a primary fallopian tube serous carcinoma.

Additionally, anti‐Ro52 antibody, which was detected in our patient, has been described to be significantly more prevalent in patients with ovarian cancer compared to in patients with colorectal cancer [20]. As paraneoplastic syndromes can precede the clinical manifestations of tubo‐ovarian cancers, accurate identification can help diagnose an underlying cancer before it is clinically apparent. In retrospect, the co‐occurrence of sensory neuropathic symptoms with anti‐Yo and anti‐Ro52 antibodies should have raised suspicion for a primary tubo‐ovarian tumor.

Furthermore, perhaps re‐biopsy of the thickened sigmoid could have been performed to provide sufficient material for IHC examination before colonic resection. This potentially could have changed the sequence of this patient's surgeries and chemotherapy. The staining pattern of our patient's sigmoid tumors showed KRT7+/KRT20– and positive PAX8 and WT1, which was highly suggestive of an ovarian origin [21, 22]. A summary of the IHC results is provided in Table 1. If sufficient material was collected for IHC examination preoperatively, we may have accurately diagnosed the patient with metastatic carcinoma of tubo‐ovarian origin initially, and the patient may have commenced neoadjuvant chemotherapy. Synchronous anterior resection, total hysterectomy, bilateral salpingo‐oophorectomy, and omentectomy could have been performed, thus preventing the need for a second operation.

**TABLE 1:** Summary of the IHC results.

	Colonic resection	Fallopian tube
KRT7	Positive	Not performed
KRT20	Negative	Not performed
CDX2	Negative	Not performed
MNF116	Positive	Positive
PAX8	Positive	Positive
WT1	Positive	Positive
ER	Positive	Positive
PR	Positive	Negative
p53	Positive	Positive
p16	Positive	Positive
SATB2	Negative	Not performed
TTF1	Negative	Not performed
GATA3	Negative	Not performed
Chromogranin	Negative	Not performed
Synaptophysin	Negative	Not performed
Calretinin	Negative	Not performed
KRT5/6	Negative	Not performed
D2‐40	Negative	Not performed

*Note:* ER/PR expression can vary between metastatic and primary foci, reflecting intratumor heterogeneity.

In conclusion, we present our lessons learned from an unusual case of HGSC of the right fallopian tube fimbriae with colon metastasis presenting with lower limb paresthesia. The presence of neurological symptoms and anti‐Yo antibodies should warrant aggressive investigation for an underlying malignancy, even when clinical or radiological signs are absent. A microscopic HGSC should be considered in female patients presenting with a cancer of unknown origin, particularly when associated paraneoplastic syndromes and antibodies are present. It is important to recognize the diagnostic utility of IHC examination and its effect on treatment decisions. As a result, re‐biopsy should be conducted if insufficient tissue was collected during the initial biopsy of a lesion.

## Author Contributions

**Tyler Ye:** writing – original draft, writing – review and editing. **Dinuke De Silva:** investigation, writing – review and editing. **Leonardo D. Santos:** investigation, writing – review and editing. **Felicia Roncolato:** writing – review and editing. **Stanley Levy:** writing – review and editing. **Auerilius Erastus Ricardo Hamilton:** conceptualization, writing – review and editing.

## Consent

Written patient consent was obtained for the publication of this case.

## Conflicts of Interest

The authors declare no conflicts of interest.

## Data Availability

Data sharing not applicable to this article as no datasets were generated or analyzed during the current study.
